# COVID-19 and antimicrobial stewardship: What is the interplay?

**DOI:** 10.1017/ice.2020.246

**Published:** 2020-05-15

**Authors:** Nikolaos A. Spernovasilis, Diamantis P. Kofteridis

**Affiliations:** 1 Internal Medicine Department, Infectious Diseases Unit, University Hospital of Heraklion, Heraklion, Greece; 2 Faculty of Medicine, University of Crete, Heraklion, Greece

*To the Editor*—Coronavirus disease 2019 (COVID-19) is currently in the spotlight, attracting all the attention of the world’s medical community. Meanwhile, other longer-term public health issues, such as antimicrobial resistance caused by misuse and/or overuse of antimicrobials, may have been relegated to the shadows. At this stage of the pandemic, and under the pressure caused by the absence of specific antivirals and vaccines, antimicrobials are being used in several ways.^1^ First, they are being repurposed for the treatment of COVID-19, as it is happening with the combination of azithromycin and hydroxychloroquine.^2^ In addition, antimicrobials are being used as empirical coverage for possible coexisting community-acquired infection of the respiratory tract, especially in severe cases of COVID-19. They are also being used as empirical coverage for possible hospital-acquired superinfection of the respiratory tract, taking into account that a significant proportion of COVID-19 hospitalized patients will have prolonged hospitalizations or will require intensive care unit admission. Furthermore, antimicrobials are being prescribed for the targeted treatment of community or hospital-acquired respiratory tract co- or superinfections. Finally, antimicrobials are being used in empirical or targeted treatment for co- or superinfection outside the respiratory tract.

However, several issues regarding the aforementioned uses of antimicrobials must be addressed. First, current clinical data on azithromycin or other antimicrobials as part of a combination for COVID-19 patients without microbial co- or superinfection are far from convincing,^3^ and high-validity studies are required to support or reject this approach.

Second, most data regarding bacterial and fungal respiratory co- and superinfections in the context of a viral respiratory pandemic are derived from previous influenza pandemics,^4^ and data from previous coronavirus epidemics are limited.^5–7^ In any case, bacterial and fungal respiratory co- and superinfections during large outbreaks of viral respiratory illnesses are probably underdiagnosed due to a shortage of trained healthcare personnel and/or supplies, infection control requirements, high workload, and the emergent nature of the primary viral disease that make the diagnosis complex and laborious. Data from the current COVID-19 pandemic, in accordance with previous coronavirus epidemics, show that the rate of bacterial and fungal co- or superinfection is relative low, but the use of broad-spectrum antimicrobials is very common.^1^ Specifically, in the current literature, bacterial and fungal co- and superinfections have been reported in 8% of COVID-19 cases, while 72% of these patients received broad-spectrum antibacterials.^1^

Finally, in the era of COVID-19, the liberal use of antimicrobials adds an unnecessary risk for possible unfavorable outcomes due to their potential toxicity.^8^ In addition, their empirical use based on local patterns of resistance has an undetermined probability of failure because during pandemics, significant changes in the pattern of endemic pathogens may evolve.^7^

Antimicrobial stewardship programs are imperative components of a successful response to COVID-19, and interventions to support the optimal use of antimicrobials are urgently needed. Until results from further research emerge and stronger evidence is available, the following antimicrobial stewardship strategies should be implemented:

- Antimicrobials should be used empirically only for severely ill COVID-19 patients, with regular reassessment of their necessity and monitoring of possible side effects.
- Empirical antimicrobial treatment must be always guided by the source of infection acquisition, local patterns of antimicrobial resistance, host factors, and concurrent drugs. However, clinicians should consider that local patterns of resistance may change during pandemics and that patient’s physiology becomes extremely altered in severe cases of COVID-19.
- Short courses of antimicrobials and early intravenous-to-oral switch whenever possible are desired; these strategies will also contribute to decreasing nurse workload and to maintaining bed capacity.
- Culture-based testing should be carried out before antimicrobials are initiated to maximize the likelihood of identifying a pathogen.
- Culture-independent techniques (eg, polymerase chain reaction or loop-mediated isothermal amplification assay) are highly important in diagnosing bacterial and fungal co- and superinfections or other viral infections during the course of COVID-19. They must be used whenever they are indicated, for example, in COVID-19 patients with severe presentation or those whose condition worsens after initial improvement.
- Procalcitonin measurements can be an important tool to reduce use of antimicrobials, but procalcitonin’s suboptimal specificity must always be considered because increased levels may occur in advanced stages of COVID-19 without proven bacterial co- or superinfection.^9^ C-reactive protein is elevated in most COVID-19 cases, and it cannot predict bacterial or fungal co- or superinfection.^10^
- Chest computed-tomography may reveal typical features of COVID-19, that is, solely ground-glass opacities, which are not common in bacterial pneumonia; therefore, such findings can help avoid antimicrobial use.

We understand the difficulties in implementing many of the aforementioned interventions in daily clinical practice during the COVID-19 pandemic. We also acknowledge that the only conclusive solution to the COVID-19 will be a safe and effective vaccine. Until then, heavy and occasionally inappropriate antimicrobial use may be observed. However, we all must look toward a post–COVID-19 era, when existing public health issues, such as antimicrobial resistance, will remain to be addressed.

## References

[1] Rawson TM , Moore LSP , Zhu N , et al. Bacterial and fungal co-infection in individuals with coronavirus: a rapid review to support COVID-19 antimicrobial prescribing. Clin Infect Dis 2020. doi: 10.1093/cid/ciaa530. PMC7197596 32358954

[2] Gautret P , Lagier JC , Parola P , et al. Clinical and microbiological effect of a combination of hydroxychloroquine and azithromycin in 80 COVID-19 patients with at least a six-day follow-up: a pilot observational study. Travel Med Infect Dis 2020. doi: 10.1016/j.tmaid.2020.101663. PMC7151271 32289548

[3] Molina JM , Delaugerre C , Le Goff J , et al. No evidence of rapid antiviral clearance or clinical benefit with the combination of hydroxychloroquine and azithromycin in patients with severe COVID-19 infection. Médecine et Maladies Infectieuses 2020. doi: 10.1016/j.medmal.2020.03.006. PMC7195369 32240719

[4] Cox MJ , Loman N , Bogaert D , O’Grady J . Coinfections: potentially lethal and unexplored in COVID-19. Lancet Microbe 2020. doi: 10.1016/S2666-5247(20)30009-4. PMC7195315 32835323

[5] Arabi YM , Deeb AM , Al-Hameed F , et al. Macrolides in critically ill patients with Middle East respiratory syndrome. Int J Infect Dis 2019;81:184–190. 30690213 10.1016/j.ijid.2019.01.041 PMC7110878

[6] Jang TN , Yeh DY , Shen SH , Huang CH , Jiang JS , Kao SJ . Severe acute respiratory syndrome in Taiwan: analysis of epidemiological characteristics in 29 cases. J Infect 2004;48:23–31. 14667789 10.1016/j.jinf.2003.09.004 PMC7127319

[7] Yap FH , Gomersall CD , Fung KS , et al. Increase in methicillin-resistant *Staphylococcus aureus* acquisition rate and change in pathogen pattern associated with an outbreak of severe acute respiratory syndrome. Clin Infect Dis 2004;39:511–516. 15356814 10.1086/422641 PMC7204093

[8] Mercuro NJ , Yen CF , Shim DJ , et al. Risk of QT interval prolongation associated with use of hydroxychloroquine with or without concomitant azithromycin among hospitalized patients testing positive for coronavirus disease 2019 (COVID-19). JAMA Cardiol 2020. doi: 10.1001/jamacardio.2020.1834. PMC7195692 32936252

[9] Lippi G , Plebani M . Procalcitonin in patients with severe coronavirus disease 2019 (COVID-19): a meta-analysis. Clin Chim Acta 2020;505:190–191. 32145275 10.1016/j.cca.2020.03.004 PMC7094472

[10] Li L , Huang Q , Wang DC , Ingbar DH , Wang X . Acute lung injury in patients with COVID-19 infection. Clin Translat Med 2020;10:20–27. 10.1002/ctm2.16 PMC7240840 32508022

